# Supplementary material for: A pathogenic CD4 T cell phenotype in experimental uveitis shares common features with other immune mediated inflammatory diseases
Source: Discov Immunol. 2025 Dec 2;5(1):kyaf019. doi: 10.1093/discim/kyaf019 (PMC13213613; doi:10.1093/discim/kyaf019)
Supplement: kyaf019_Supplementary_Data [file kyaf019_supplementary_data.pdf]

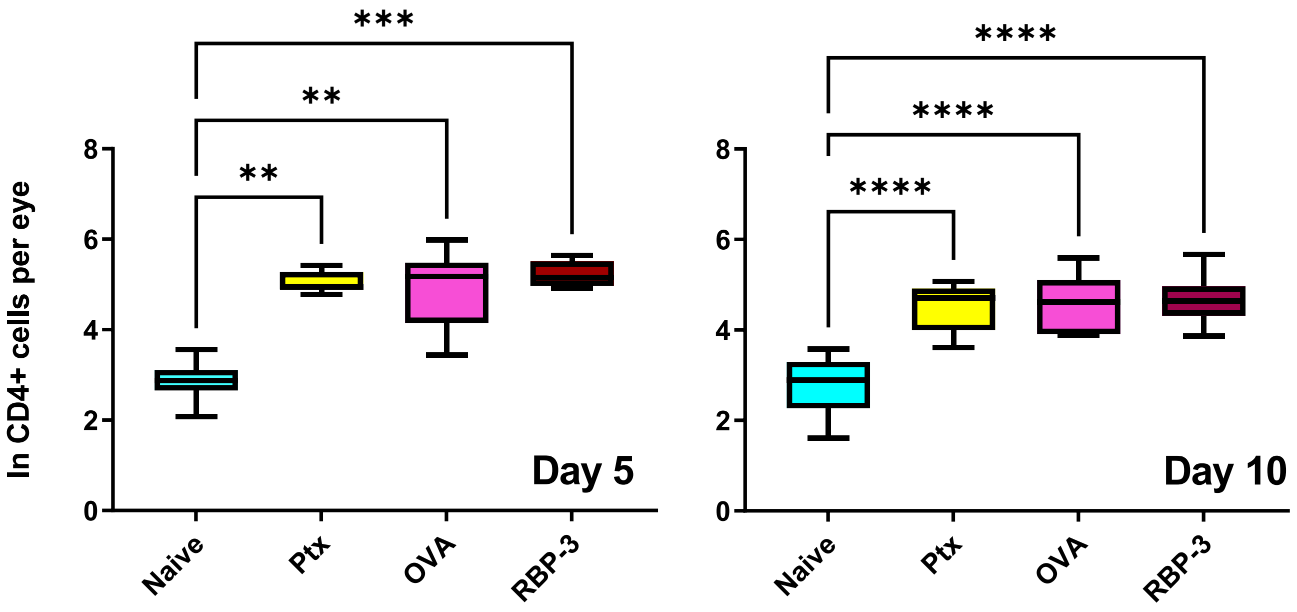

Fig. S1: Immunisation with pertussis alone (Ptx) or with peptides (OVA or RBP-3 1-20) emulsified in CFA leads to a significant increase in CD4 cell numbers in the eye, greater on day 5 compared with day 10.

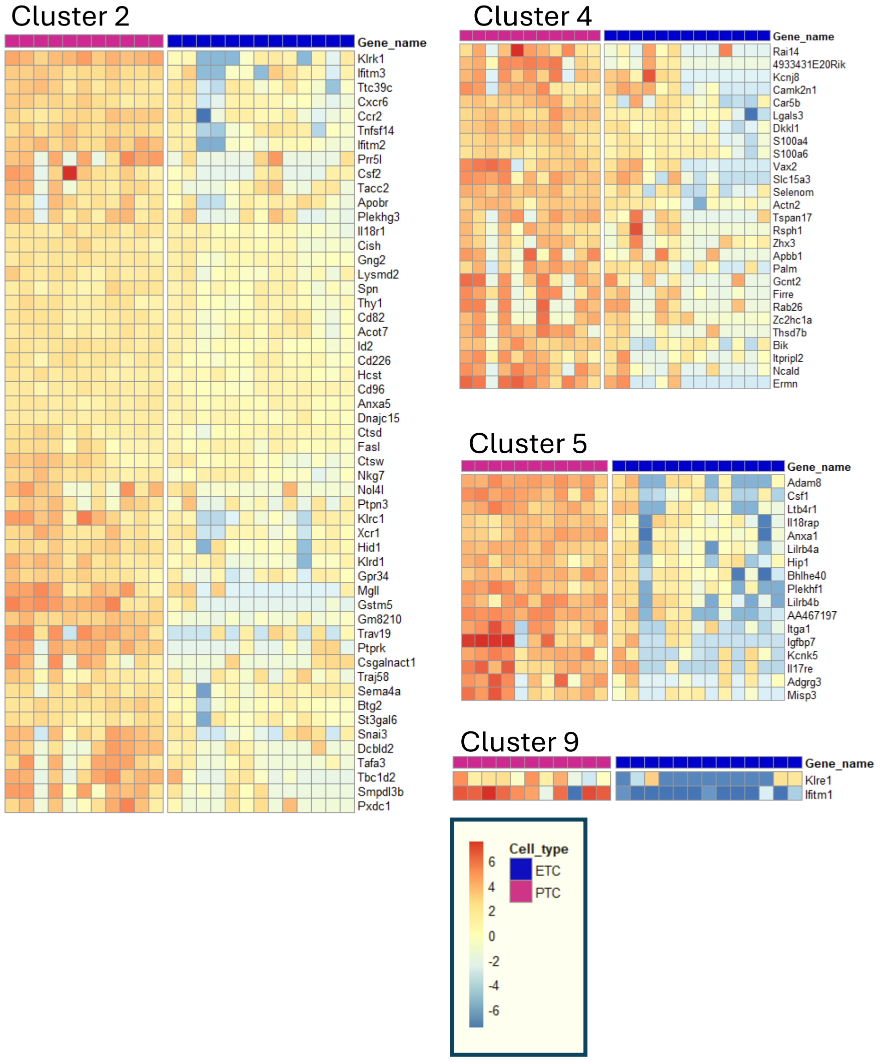

Fig. S2. Genes upregulated in pathogenic cells grouped by k-means clustering

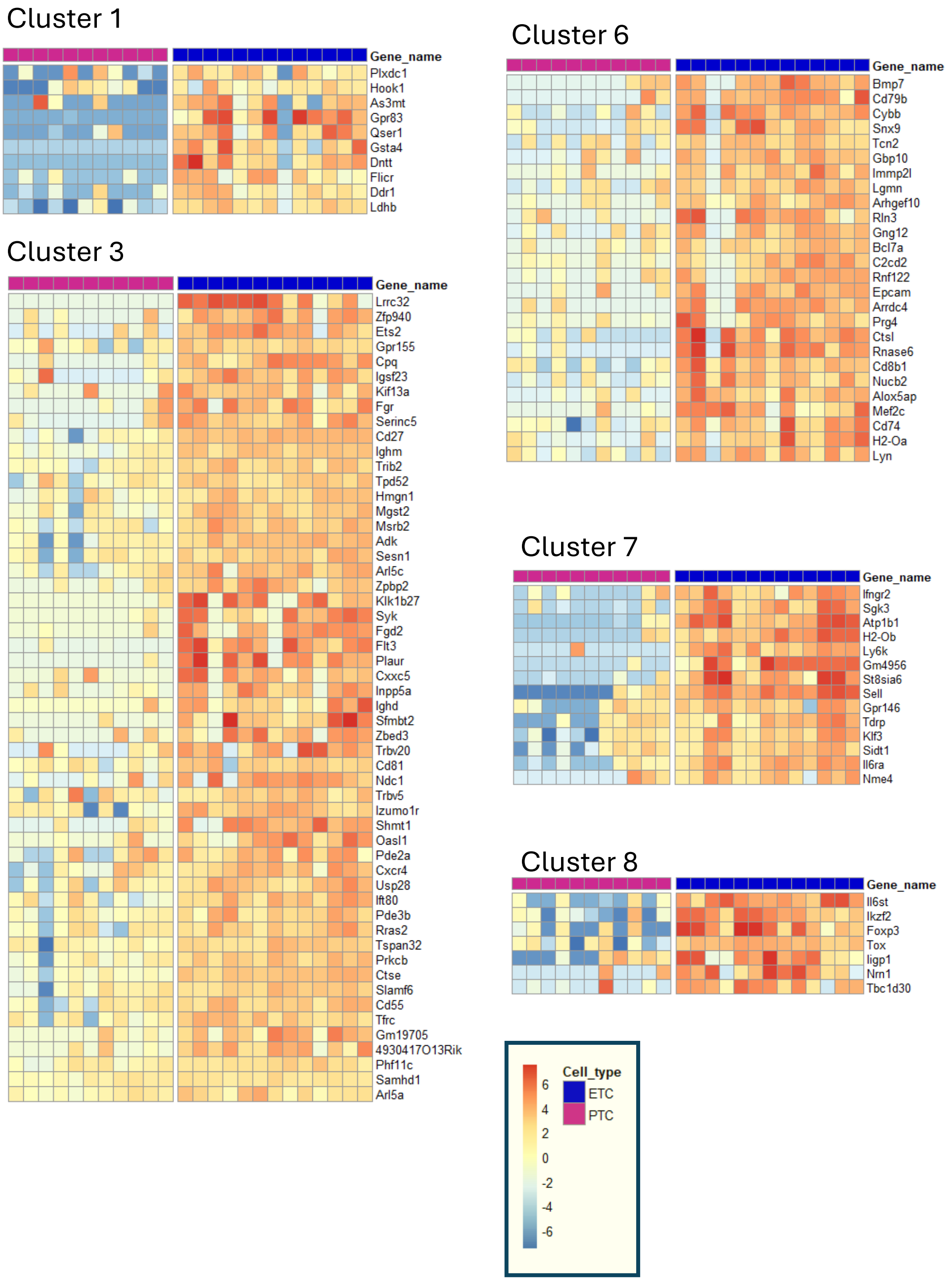

Sup Fig. 3. Genes downregulated in pathogenic cells grouped by k-means clustering

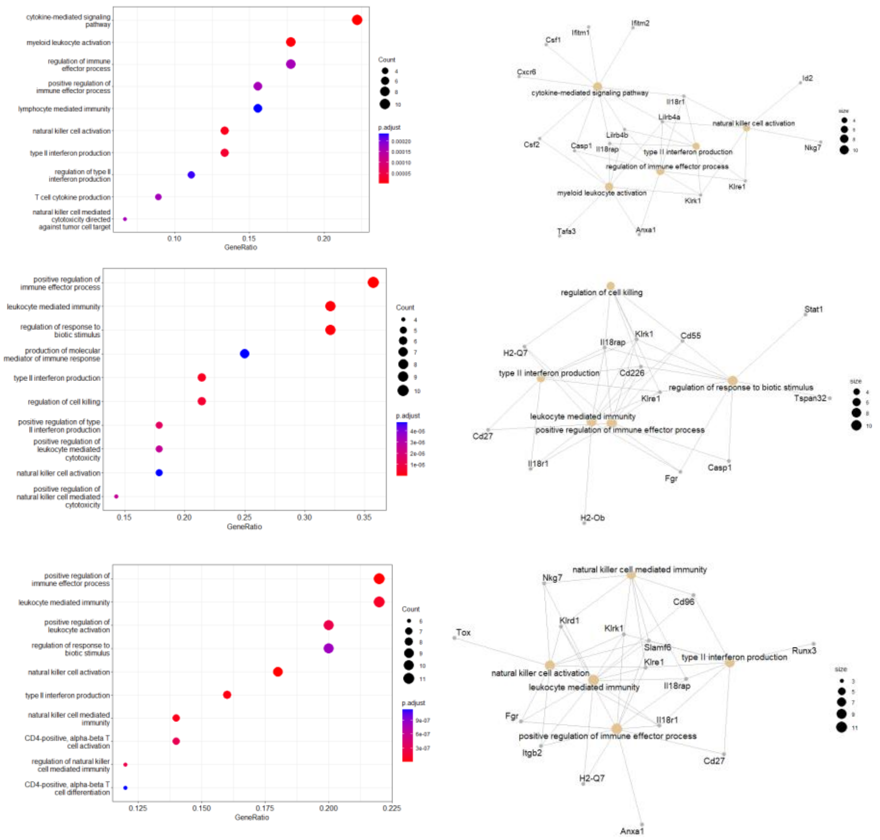

Sup Fig. 4. Analysis of leading edge genes from GSEA analysis of the CD4 pathogenic phenotype in active EAU and NKG2D knockouts
